# Risk factors for epiretinal membrane surgery after initial pars plana vitrectomy for rhegmatogenous retinal detachment

**DOI:** 10.20407/fmj.2020-027

**Published:** 2021-03-20

**Authors:** Yuki Takamidou, Tadashi Mizuguchi, Ryouta Sakurai, Mitsuo Sugimoto, Atsuhiro Tanikawa, Masayuki Horiguchi

**Affiliations:** Department of Ophthalmology, Fujita Health University, School of Medicine, Toyoake, Aichi, Japan

**Keywords:** Epiretinal membrane surgery, Pars plana vitrectomy, Rhegmatogenous retinal detachment, Retinal detachment, Risk factors

## Abstract

**Objectives:**

The purpose of this study was to examine the incidence of, and risk factors for, epiretinal membrane (ERM) surgery after an initial pars plana vitrectomy (PPV) for rhegmatogenous retinal detachment (RRD).

**Methods:**

The records of consecutive patients (3,495 eyes of 3,387 patients) who underwent RRD repair at Fujita Health University Hospital between January 1, 2008, and February 28, 2019, were retrospectively reviewed. A total of 1,736 eyes without an ERM in preoperative optical coherence tomography were included in this study.

**Results:**

The incidence of ERM surgery after RRD repair was 2.4%. The mean time from RRD repair to ERM surgery was 19.5±27.2 months. The odds ratios after adjusting for age and sex were as follows: the preoperative visual acuity (logarithm of the minimum angle of resolution, logMAR), 2.17 (p=0.02; 95% confidence interval [CI], 1.11–5.16); axial length, 1.38 (p=0.002; 95% CI, 1.12–1.72); 20-gauge vitreous surgery instruments, 3.82 (p<0.0001; 95% CI, 2.02–7.16); internal limiting membrane (ILM) peeling, 0.28 (p=0.033; 95% CI, 0.05–0.92). ERM surgery improved visual acuity from 0.36 to 0.01 logMAR, even at ≥1.5 years after RRD repair.

**Conclusions:**

Careful follow-up is required in the following cases: long axial length before RRD repair, low visual acuity, use of 20-gauge vitreous surgery instruments, and a lack of ILM peeling.

## Introduction

One possible complication after initial surgery for rhegmatogenous retinal detachment (RRD) is epiretinal membrane (ERM) formation, which leads to metamorphopsia and deterioration of visual acuity (VA).^1–3^ The growth of retinal pigment epithelium, originating from retinal tears in the macula, leads to the proliferation of various cells, such as retinal glial cells, contributing to ERM formation.^4–7^

According to previous studies, the incidence of ERM formation after pars plana vitrectomy (PPV) for retinal detachment (RD) ranges from 4% to 15%,^8–12^ and the incidence of ERM surgery ranges from 2.8% to 4.3%.^8–12^ This variability in the incidence of ERM surgery depends on the study design. For example, some studies used optical coherence tomography (OCT),^8,10,12^ whereas other studies did not.^9,11,13^ Furthermore, some studies included patients with proliferative vitreoretinopathy (PVR) who went on to have ERM surgery,^11^ whereas other studies excluded PVR patients.^8–10,12,13^ There are many studies that did not exclude patients who had an ERM before RRD repair;^8,9,13^ in other words, those studies may have included patients who originally had an ERM.

Based on various large epidemiologic studies, the major risk factors for ERM reportedly are older age^14–22^ and female sex.^17,23^ In the Japanese, population-based Funagata Study,^20^ the prevalence rate for an ERM was 5.7%, which was similar to that in Caucasian populations.^23^ Only increasing age was consistently associated with ERM formation in that study. According to a recent epidemiological study of ERMs (Jianging Eye Study^24^) using OCT, the incidence of ERMs in individuals 50-years-old and over was 8.4%, and the risk factors were age and female sex. In other words, older age and sex should be considered in studies of PPV for RRD repair as well.

The primary purpose of this study was to examine the incidence of, and risk factors for, ERMs in patients without an ERM on OCT before their initial surgery for RRD, after adjusting for age and sex. The secondary purpose was to evaluate the time from RRD repair to ERM peeling, and the changes in VA from before to after ERM surgery .

## Methods

### Study subjects

**Figure 1:**
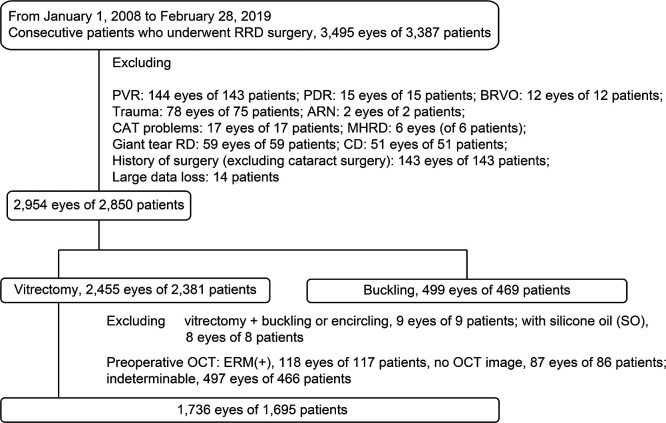
Inclusion and exclusion criteria

The electronic medical records of consecutive patients who underwent RRD repair at Fujita Health University Hospital between January 1, 2008, and February 28, 2019, were retrospectively reviewed. Of the 3,387 consecutive patients (3,495 eyes) who underwent RRD repair, patients were excluded from this study if they had a history of: disease with a high risk of tractional RD (proliferative diabetic retinopathy: 15 eyes of 15 patients); branch retinal vein occlusion (12 eyes of 12 patients); acute retinal necrosis (2 eyes of 2 patients); complications of cataract surgery within the previous year (17 eyes of 17 patients); PVR of grade C or higher (144 eyes of 143 patients); RD that caused a macular hole (6 eyes of 6 patients); or a giant tear RD (59 eyes of 59 patients). We also excluded eyes complicated by choroidal detachment (51 eyes of 51 patients), and eyes with a history of eye surgery including RD repair, excluding cataract surgery (143 eyes of 143 patients), as well as patients with substantial missing data (14 patients) (Figure 1). After those exclusions, 2,954 eyes (of 2,850 patients) remained.

The types of surgery performed in the remaining eyes were: vitrectomy (2,455 eyes of 2,381 patients) and buckle surgery (499 eyes of 469 patients). From the 2,455 eyes that underwent vitrectomy, we further excluded eyes with: PPV with buckling or encircling (9 eyes of 9 patients); PPV with silicone oil tamponade (9 eyes of 9 patients); eyes with an ERM that was detected by preoperative OCT (118 eyes of 117 patients); eyes with an ERM that could not be detected by OCT (497 eyes of 466 patients); and those with no preoperative OCT data (87 eyes of 86 patients) (Figure 1). Therefore, a retrospective analysis of 1,736 eyes of 1,695 patients was performed.

This study was performed in accordance with the Declaration of Helsinki, after approval by the Institutional Review Board of Fujita Health University (approval number: HM19-099). Opt-out informed consent, and consent for participation and publication of data were obtained from all study participants.

### Surgical protocol

Four surgeons (TM, AT, MS and MH) performed 20-, 23- and 25-gauge transconjunctival vitrectomies. The Accurus Vitrectomy System (Alcon Laboratories, Fort Worth, TX, USA), the Alcon Constellation^®^ system (Alcon Laboratories) and the EVA^®^ vitrectomy system (DORC International, Zuidland, The Netherlands) were used for vitreous surgery. A wide-angle viewing system (Optical Fiber-Free Intravitreal Surgery System [OFFISS^®^]; Topcon, Tokyo, Japan) was used as a microscope. Simultaneous cataract surgery was performed at the discretion of the operating surgeon. The surgeons performed complete PPV with the induction of posterior vitreous detachment using a triamcinolone acetate stain. Subretinal fluid was drained from existing retinal tears or intentional retinal breaks (RBs). In some cases, for balloon RDs, the detached retina was flattened with perfluorocarbon liquid. Endophotocoagulation or cryopexy was used for retinopexy of small areas (e.g., retinal tears or lattice degeneration). After a complete fluid-air exchange procedure, vitrectomy with air, 20% sulfur hexafluoride (SF6) gas or 12% perfluoropropane (C3F8) gas tamponade was performed. Intraocular chandelier lighting was used as an option. In most cases, patients were asked to adopt appropriate head positions (e.g., face down position) after surgery.

### ERM evaluation

Both fundus images and OCT (OCT3000 [Carl Zeiss Meditec, Inc., Dublin, CA, USA], OCT-1 [Carl Zeiss Meditec, Inc.] or DRI OCT [Topcon, Tokyo, Japan]) were used to evaluate ERMs. To evaluate the presence or absence of an ERM, all OCT and fundus images taken prior to RRD repair were examined by five reviewers (one-fifth of images, randomly assigned, were reviewed by each of TM, YT, MS, AT and MH). When there was no consensus among the five reviewers, disagreement was resolved by discussion between two reviewers (TM and YT). The following were excluded from the analysis: patients without an ERM that could be detected by OCT, because of the turbidity of the optic media caused by vitreous hemorrhage (VH); patients with low-resolution images of the fovea, due to macular detachment; and patients without an ERM that could be detected by OCT for other reasons (Figure 1).

In the case of deterioration of VA or worsening of metamorphopsia after RRD repair, ERM surgery was considered by follow-up doctors. Patients were then referred back to our hospital for ERM surgery. ERMs were treated by ERM peeling with a standard 3-port PPV and intraocular forceps. The internal limiting membrane (ILM) was also peeled using indocyanine green and Brilliant Blue G (Sigma-Aldrich).

We reviewed the charts for incidence and predictor factors for ERM surgery after PPV for repair of primary RRD. The following details were recorded: factors measured in their PPV preoperative examination (i.e., time from onset to surgery, lens status, BCVA, axial length, percentage of macula-off cases, number of quadrants with an RD, number of total RBs), and operative procedure (i.e., percentage of cases with simultaneous cataract surgery, type of gas tamponade substance, gauges of surgical instruments, number of cryopexies, number of cases with endophotocoagulation, percentage with ILM peeling, and percentage with 360-degree laser retinopexy, operation time) (Table 1).

### Statistical analyses

All statistical analyses (Chi-square test, Fisher exact test, paired t-test, Wilcoxon rank sum test and multivariate regression analysis) were performed, as appropriate, to estimate the odds ratio after adjusting for patient age and sex, and 95% confidence intervals (CIs) were calculated, using JMP ver. 9.0 (SAS Institute Inc., Cary, NC, USA). In addition, multivariate analysis with added variables (such as operation time, which were considered to be a confounding factor per test) was performed, after adjusting for age and sex. P<0.05 was considered statistically significant.

## Results

**Figure 2:**
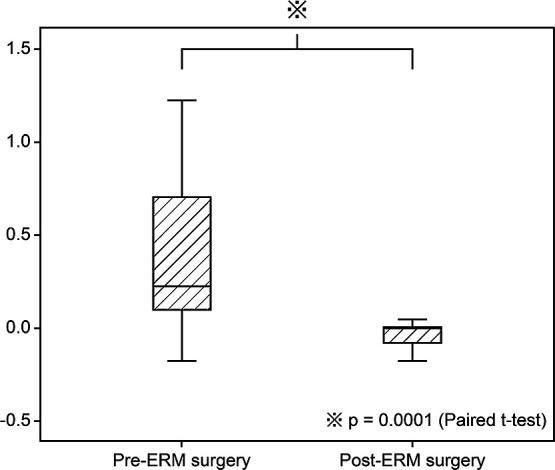
Box plot showing the comparison between best-corrected visual acuity (logMAR) values before and after epiretinal membrane (ERM) surgery

Table 1 shows the characteristics of the 1,736 eyes of 1,695 patients with successful retinal reattachment after PPV. The mean age at onset of RRD was 56.2 years, and RRD was more common in males (68.8%) than females. The mean time from RRD onset to surgery was 9.1 days, the percentage of macula-off RDs was 40%, the mean number of RD quadrants was 1.7, and the mean total number of RBs per eye was 2.5. In the present study, there were 41 patients who underwent ERM surgery, so the incidence of ERM surgery after initial PPV for RRD was 2.4%; the mean time from initial PPV to ERM surgery was 19.5±27.2 months. ERM surgery significantly improved VAs, from 0.36±0.38 to 0.01±0.22 logMAR (p=0.0001, paired t-test) (Figure 2).

We calculated the odds ratios associated with ERM surgery after adjusting for age and sex (Table 2). In this study, the risk factors for ERM surgery after PPV for RRD were as follows: axial length, preoperative VA, use of 20-gauge vitreous surgery instruments, and the absence of ILM peeling (Table 2).

### Preoperative VA

The odds ratio for preoperative VA was 2.17 (p=0.02; 95% CI, 1.11–5.16), indicating preoperative VA as a risk factor. The odds ratio for preoperative VA, after adjusting for age, sex and incidence of macular detachment, was 2.46 (p=0.03; 95% CI, 1.07–6.87). The higher the preoperative logMAR (i.e., the lower the preoperative visual acuity), the higher the risk of ERM.

### Axial length

The odds ratio for ocular axial length was 1.38 (p=0.002; 95% CI, 1.12–1.72), suggesting ocular axial length as a risk factor. With an ocular axis increment of 1 mm, the risk of ERM increased by 38%.

### Gauge size of the vitreous surgery instruments

The odds ratio for 20-gauge, but not 23- nor 25-gauge, surgical instruments was 3.82 (p<0.0001; 95% CI, 2.02–7.16), indicating a risk factor for ERM surgery. In addition, the odds ratio for 20-gauge instruments, after adjusting for the duration of the original PPV, was 3.99 (p<0.0001; 95% CI, 2.12–7.46), indicating that surgery with 20-gauge instruments was a risk factor for ERM surgery, regardless of operation time.

### ILM peeling

The odds ratio for ILM peeling was 0.28 (p=0.033; 95% CI, 0.05–0.92), showing that ILM peeling significantly reduced the incidence of ERM surgery. In addition, because the presence or absence of macular detachment was considered to be a confounding factor for ILM peeling, the odds ratio, after adjusting for age, sex and the presence or absence of macular detachment, was also calculated. The odds ratio was 0.27 (p=0.03; 95% CI, 0.04–0.90), indicating that ILM peeling reduced the incidence of ERM surgery, regardless of the presence of macular detachment.

## Discussion

In the present study, the incidence of ERM surgery (primary PPV) after RRD repair was 2.4%. The mean time from RRD repair to ERM surgery was 19.5±27.2 months. The incidence of ERM surgery in this study was lower than that in previous studies.^8–10^ Our study is novel in that, among other reasons, it examined differences according to the gauge of the vitrectomy instrument. Moreover, to our knowledge, this is the first study to recognize axial length as a risk factor for ERM surgery after RRD repair.

However, in a previous report,^10^ when patients whose ERM was detected using swept-source OCT before RRD repair were excluded, as in the present study, the incidence of ERM surgery was 2.8% and the mean time from RRD repair to ERM surgery was 5.0 months. The incidence of ERM surgery and the median time from RRD repair to ERM surgery in this study were equivalent to those in the study by Ishida et al.^10^ However, the long duration from RRD repair to ERM surgery (19.5 months) in this study was due to a longer follow-up period.^10^

### Risk factors for ERM

Univariate analyses in previous studies identified the following as the risk factors for ERM after PPV: multiple retinal tears,^8^ large RBs,^8,11^ VH,^10^ macular detachment,^9^ pseudophakia and aphakia.^11,12^ In this study, multiple retinal tears, VH, macular detachment, pseudophakia and aphakia were not identified as risk factors for ERM after PPV, and the size of the retinal tears was not examined. There are two possible reasons for multiple retinal tears, macular detachment and VH being not identified as risk factors for ERM surgery in the present study. First, as the time from RRD onset to RRD repair was 9.1 days, the period during which pigment epithelial cells and proliferating cells were scattered was limited. Second, regarding the PPV surgical procedure, in most cases, during the fluid-air exchange procedure, pigment epithelial cells and proliferating cells were removed by the surgeon, by creating an intentional retinal tear in the posterior pole, to completely drain the subretinal fluid under the macula. Eyes that were not suitable for OCT evaluation before RRD repair were excluded from the study because of the turbidity of the optic media caused by VH and high macular detachment. Therefore, the incidence of ERM surgery due to VH and macular detachment may have been underestimated.

In this study, the risk factors for ERM surgery after PPV for RRD were axial length, preoperative VA, use of 20-gauge surgical instruments and the absence of ILM peeling. Preoperative VA, regardless of the incidence of macular detachment, was a risk factor; this result differs from those in previous studies.^8–13^

The finding that axial length was a risk factor also differs from results in previous studies.^8–13^ In the present study, ocular axis increments of 1 mm increased the risk of ERM by 38%. A large-scale study of idiopathic ERM^25^ also identified a long axial length as a risk factor. The longer the axial length, the stronger the tangential traction of the vitreous body on the retina at the macula.

The use of 20-gauge vitreous surgery instruments was a risk factor for ERM surgery, regardless of the operation time. To our knowledge, there have not been any reports that examined the difference in the gauge size of the vitreous surgery instruments as a risk factor for ERM surgery after PPV for RRD repair.

Previous randomized trials comparing 20- and 23-gauge vitreous surgery reported chronic inflammation after 20-gauge vitreous surgery.^25^ In a study of ERM after PPV for RRD using 23-gauge instruments, Moon et al.^8^ concluded that sutureless surgery using 23-gauge instruments effectively reduced the incidence of ERM, relative to that in previous studies of ERM after PPV for RRD.^8^ Surgery using 20-gauge instruments required sutures in all patients in this study as well: some patients who underwent surgery using 23- or 25-gauge instruments also required sutures. Therefore, it is not known whether suture-related inflammation affected the outcome.

There are many studies showing that peeling of the ILM, which is the basement membrane of the Müller cells and a scaffold for cell proliferation, reduces ERM formation after RRD repair.^26–29^ In this study, ILM peeling significantly reduced the incidence of ERM surgery, regardless of the presence of macular detachment, consistent with previous studies.^26–29^

In this study, there were two cases of ERM surgery after initial PPV for RRD with ILM peeling. After confirming these two surgical records and video, the ILM was visualized using Brilliant Blue G in both cases, and it was confirmed that the ILM was peeled off completely. However, ILM peeling leads to ERM formation in the macular ring, resulting in metamorphopsia and low VA. ILM peeling of a larger area may not have been required in these two patients.

We previously reported a study on RD in patients with high myopia, wherein we measured the axial length and vitrectomized space.^29^ We measured the volume of space replaced by air during the fluid-air exchange in the vitrectomy (vitrectomized space) for intravitreal gas injection. In future studies, we hope to examine eyeball morphology, such as the axial length and vitrectomized space, with regard to the risk of ERM surgery after RRD repair.

### Limitations

This study has some limitations. This was a retrospective study and the patients were followed by our hospital, related hospitals or clinics that referred the patients to our hospital. The indication criteria for ERM surgery should be strict. However, there were no clear criteria for surgery, so surgical decisions were left to the discretion of the follow-up doctor. Therefore, the incidence of ERMs and the resulting ERM surgery rate are unknown.

Not all variables were included in the multivariate analysis because of the small sample of patients who underwent ERM surgery. Therefore, age- and sex-adjusted odds ratios were calculated. In addition, the multivariate analysis with an added variable per test (variables considered as confounding factors, such as operation time) was performed after adjusting for age and sex. However, the strength of our study was that it used multiple regression analysis for the adjustment by age and sex, excluded ERM by preoperative OCT, and had a long follow-up period with many participants.

The incidence of ERM surgery after initial PPV for RRD repair is not very high. Moreover, careful follow-up is required in the following RRD-repair cases: long axial length, high preoperative VA, use of 20-gauge instruments and absence of ILM peeling. These findings suggest that ERM surgery significantly improves VA more than 1.5 years after RRD repair. Nevertheless, further prospective studies are needed to confirm our results.

## Figures and Tables

**Table1:** Characteristics of the participants, and their PPV surgeries for RRD

Characteristics	
Age	56.2±10.7
Male/female (male%)	68.8
Phakia (%)	85.7
Aphakia (%)	0.8
IOL (%)	13.5
Time from onset to surgery (day)	9.1±15.1 (median, 5)
Axial length	25.8±1.8
BCVA (logMAR)	0.38±0.62
Macular off (%)	40
Number of quadrants with RD	1.7±0.8
Number of total RBs	2.5±1.9
Number of RBs in the superior quadrant	1.3±1.4
RBs in superior RD (%)	62.3
PVD (%)	93.4
Vitreous hemorrhage (%)	5.9
Characteristics	
Vitrectomy+PEA+IOL (%)	69.8%
Vitrectomy+PEA (%)	0.9
Vitrectomy	29.3
SF6 (%)	89.6
C3F8 (%)	4
Air (%)	6.3
20 G (%)	19.5
23/25 G (%)^†^	80.3
Cryopexy (%)	31.6
Number of cryopexy	2.4±4.8
Number of PC	606.1±427.2
360-degree laser retinopexy (%)	7.9
ILM peeling (%)	15.2
Operation time (min)	58.6±21.5

^†^ 23/25G is sum of proportion of 23 and 25 gauge size of the vitreous surgery instruments PPV, pars plana vitrectomy: RRD, rhegmatogenous retinal detachment: IOL, Intraocular lens: BCVA, best corrected visual acuity: logMAR, logarithm of the minimum angle of resolution: RD, retinal detachment: RBs, retinal breaks: PVD, Posterior vitreous detachment. PEA, phacoemulsification: SF6, sulfur hexafluoride: C3F8, perfluoropropane: G, gauge size of the vitreous surgery instruments: PC, endophotocoagulation: ILM, Internal limiting membrane.

**Table2:** Risk factors (odds ratio and 95% CI) for ERM surgery after RRD repair (odds ratios after adjusting for age, sex and 95% CI)

	Odds ratio	P value	95% CI
Age	1.02	0.1782	0.99–1.05
Male/female (male %)	1.31	0.422	0.67–2.46
Time from onset to surgery (day)	1.01	0.307	0.99–1.07
Axial length (mm)	1.38	0.0021*	1.12–1.72
BCVA (logMAR)	2.17	0.0205*	1.11–5.16
Macular off	1.41	0.3003	0.74–2.87
Number of quadrants with RD	1.53	0.5887	0.34–7.91
Superior RD	1.07	0.8416	0.57–2.08
Number of total RBs	0.98	0.8215	0.84–1.17
Number of RBs in superior quadrant	0.94	0.5374	0.78–1.17
RBs in the superior RD	1.07	0.4222	0.67–2.46
PVD	1.04	0.9443	0.36–4.47
Vitreous hemorrhage	1.8	0.3073	0.53–4.63
Vitrectomy+PEA+IOL	1.09	0.1818	0.80–3.94
Air/SF6 or C3F8	1.31	0.4078	0.68–2.48
20 G/23 or 25 G	3.82	P<0.0001*	2.02–7.16
Cryopexy	1.21	0.562	0.62–2.30
Number of cryopexy	1	0.8718	0.13–21.6
Number of PC	0.22	0.1381	0.04–1.07
360-degree laser retinopexy	1.98	0.1627	0.73–4.48
ILM peeling	0.28	0.033*	0.045–0.92
Operation time (min)	2.34	0.4798	0.24–29.4

* P<0.05 CI, confidence interval: ERM, epiretinal membrane: RRD, rhegmatogenous retinal detachment: BCVA, best corrected visual acuity: logMAR, logarithm of the minimum angle of resolution: RD, retinal detachment: RBs, retinal breaks: PVD, Posterior vitreous detachment: PEA, phacoemulsification: IOL, intraocular lens: SF6, sulfur hexafluoride: C3F8, perfluoropropane: G, gauge size of the vitreous surgery instruments: PC, endophotocoagulation: ILM, Internal limiting membrane.
